# Social representations of male circumcision as prophylaxis against HIV/AIDS in Zimbabwe

**DOI:** 10.1186/s12889-015-1967-z

**Published:** 2015-07-02

**Authors:** Antony Chikutsa, Pranitha Maharaj

**Affiliations:** School of Built Environment and Development Studies, University of KwaZulu Natal, Durban, South Africa; Department of Development Studies, Zimbabwe Open University, Harare, Zimbabwe

**Keywords:** Male circumcision, Social representations, HIV prevention

## Abstract

**Background:**

The World Health Organisation recommended the scale-up of voluntary medical male circumcision (VMMC) as an additional HIV prevention method in 2007 and several countries with high HIV prevalence rates including Zimbabwe have since adopted the procedure. Since then researchers have been preoccupied with establishing the level of knowledge and acceptability of circumcision in communities that did not traditionally circumcise. Despite evidence to suggest that knowledge and acceptability of voluntary medical male circumcision is high, there is also emerging evidence that suggest that uptake of circumcision among men has been below expectations. The purpose of this study was thus to investigate people’s representations of male circumcision that may influence its uptake.

**Methods:**

Data for this study was collected through focus group discussions with men and women aged between 18 and 49 years. This age group was selected because they are still very sexually active and are within the target population of the upscale of voluntary medical male circumcision programme. Women were included in the study because they would be directly involved in a decision to have their son(s) get circumcised for HIV prevention. The study was carried out in Harare, Zimbabwe. Obtained qualitative data was analysed using thematic content analysis.

**Results:**

Results suggest that circumcision is perceived as an alien culture or something for “younger” men or “boys” who are not yet married. The findings also suggest that there are beliefs that circumcision maybe associated with satanic rituals. The issue of condom use after circumcision was also discussed and it was found that some men do not see the need for using condoms after getting circumcised.

**Conclusions:**

There is an urgent need for the development of communications that directly address the misconceptions about voluntary medical male circumcision. There is need for communication that encourages circumcised men to continue using condoms.

## Background

It is now generally accepted in public health spheres that medical male circumcision is efficacious in the prevention of HIV infection. This follows evidence from randomised controlled trials in South Africa, Uganda and Kenya which showed that circumcision has a protective effect of up to 60 % on men [1–3]. These findings had a profound impact on the drive to scale up male circumcision for HIV prevention, and fuelled subsequent debates on the mechanisms of protection, safety outcomes, accessibility and costs, and acceptability of the procedure to non-circumcised communities [4–10]. Male circumcision has also been linked to reduced chances of getting infected with genital herpes, bacterial vaginosis, trichomoniasis, and penile and cervical cancer [11].

Zimbabwe adopted male circumcision for HIV prevention in 2009 as an additional HIV prevention strategy to augment HIV prevention programmes that were based on the ABC Model which advocates the promotion of abstinence, faithfulness, and the consistent and correct use of condoms. Since then, there has been a vigorous campaign to encourage men, especially those between the ages of 13 and 29 years, to get circumcised [12]. The Zimbabwe Demographic and Health Survey of 2006 found an average male circumcision prevalence of about 9.2 % [13] while another study found a male circumcision prevalence of 20 % [14]. This suggests that male circumcision is not a common practice in Zimbabwe. Despite the low prevalence, evidence suggests that there is support for the roll-out of male circumcision for HIV prevention [14, 15]. Studies on male circumcision have focused on the acceptability of male circumcision and the factors that may affect the favourable reception of this addition HIV prevention strategy with little attention being given to the meanings that people attach to circumcision. Just like in the case of condoms, socially constructed meanings on voluntary medical male circumcision (VMMC) may provide a deeper understanding of the factors influencing VMMC uptake.

Studies on medical male circumcision have mainly focused on acceptability of circumcision as an HIV prevention method in communities that do not traditionally circumcise. In addition to assessing the acceptability of medical male circumcision, these studies also attempted to document possible barriers to the promotion of the procedure as a prophylaxis against HIV. In a study to assess the acceptability of male circumcision among the Luo in Kenya, it was observed that the primary barriers to male circumcision were cultural identification, fear of pain and excessive bleeding and costs [16]. They also noted that the clinicians lacked the knowledge and the resources to offer safe circumcision counselling and services. Generally, they concluded that acceptability was higher than was previously suspected. A similar study was done again in Kenya a year after the launch of the male circumcision roll-out programme and the findings suggested the need to dispel misconceptions about male circumcision [9]. Furthermore, the study recommended the involvement of religious leaders, women’s groups, and making circumcision relevant to men who are already practicing an HIV prevention method. Acceptability studies were also done in South Africa by Scott, Weiss and Viljoen [17] and by Lissouba, Taljaard et al*.* [18]. Both studies reported fairly high acceptability levels for both infant and adult male circumcision for HIV prevention. Similarly supportive findings were reported by Lukobo and Bailey [19] in Zambia and by Mavhu et al*.* in Zimbabwe [14].

In addition to evaluating the acceptability of male circumcision and barriers to uptake, studies have also reviewed safety outcomes of circumcision. In 2010, Perera et al*.* did a systematic review of literature on randomised controlled trials specifically focussing on safety and efficacy of non-therapeutic medical male circumcision [20]. They observed that medical male circumcision is generally safe with few studies reporting adverse events after the procedure. Weiss et al*.* also did a systematic review of literature on complications associated with neonatal medical male circumcision and concluded that studies report very few severe complications except in cases where the procedure is undertaken at older ages, by inexperienced providers or in non-sterile conditions [21].

Despite these advances in knowledge about male circumcision for HIV prevention, few studies have been done that focus on meanings that people attach to circumcision. As programme evaluation evidence on male circumcision roll-out campaigns trickle in, it is becoming clearer that knowledge and acceptability alone are not sufficient to trigger demand for voluntary medical male circumcision (VMMC) services. In Zimbabwe, for example, Hatzold et al*.* reported that despite rapid scale-up of service provision, uptake of VMMC has been slower than anticipated [12]. This therefore calls for a shift in perspective on the roll-out of VMMC for HIV prevention to focus on the symbols and socially constructed meanings that are attached to circumcision. The study of symbols and meanings was crucial in understanding the failure of the condom as a contraceptive method and as a protective method for STIs and HIV, particularly among couples in marital unions [22–24].

The study of symbolic meanings of circumcision is not entirely new as many scholars over the centuries have attempted to understand the religious and cultural meanings of circumcision. Mbiti, for example, noted that male circumcision rituals, like all other rituals in traditional African religion that involve the public shedding of blood and sacrifice, were designed to bring oneness between the individual, his ancestral land and hence the ancestors [25]. Among the Kurya of Tanzania, Mshana et al*.* [26] noted that male circumcision is a rite of passage from childhood to adulthood. When a boy is ushered into adulthood through circumcision, he is then allowed to assume family responsibilities and to be a ‘guardian’ of tribal secrets. Over and above the power that the ritual confers on individuals, circumcision is important for social organisation and cultural identity. In addition, the practice gives Kurya leaders symbolic power and recognition. Among the Xhosa of South Africa, male circumcision is the only recognised way that a boy is transformed into a man thus gaining access to resources and other responsibilities. An uncircumcised man among the Xhosa is treated like a child and is not allowed to marry or to sacrifice to ancestral spirits [27]. Also, a man who obtains a circumcision through other means other than through the traditional ceremony is considered as a social outcast. According to Vincent, male circumcision among the Xhosa is also intimately linked to the desire for identity with kinsmen [28]. Among the Aborigines of Australia, Lawlor [29] noted that male circumcision rituals symbolise the separation of a boy from maternal care thus ushering him into a world of adult self reliance. In other words, initiation symbolises death and resurrection. Circumcision among the Aborigines is believed to break the boy’s connection with the feminine world and introduce him to an expanded consciousness and a new awareness of the spiritual realm. This view of separating feminine tendencies is also supported by Hosken [30] who postulated that the prepuce of the penis represents femininity in a boy. Thus, circumcision serves to establish the correct sex of a person in society. In Cameroon among the Nso, circumcision rituals are associated with fertility and sexuality [31]. It is believed that circumcision prepares the penis for coitus and reproduction. An uncircumcised man is viewed as immature and inclined to poor sexual and reproduction performance. Hellsten also observed that circumcision among this ethnic group is viewed as a way of taming and restraining sexual desires thus helping men to act more responsibly [31]. Despite the detailed literature on symbolism in religious and traditional circumcision, studies that focus on meanings of voluntary medical male circumcision for HIV prevention, particularly in communities that do not traditionally circumcise, are still very limited. The purpose of this paper is thus to contribute to the ongoing debate on male circumcision.

### Theoretical framework

This paper draws on the social representations approach to understand the social and cultural meaning of male circumcision as an HIV prevention method. Moscovici defined social representations as a contemporary version of commonsense [32]. In particular, social representations are a set of concepts, statements and explanations originating in daily life in the course of inter-individual communications. Winskell et al*.* defined social representations as culturally shared mental phenomena that communicate norms and values in symbolic form [33]. They further elaborate that social representations are often pre-conscious and therefore less subject to informant bias than conscious evaluative judgements like attitudes. Maurya defined social representations simply as commonsense theories generated by people to understand everyday reality [34].

Social representations have four main functions [35]. Firstly, they have a knowledge function through which reality is understood and explained. Secondly, social representations situate individuals and groups in a social field and enable the development of a social identity compatible with the norms and values of society. Thirdly, social representations have a guidance function through which behaviour and practices are measured. Finally, social representations have a justificatory function which permits after-the-fact justification of positions and behaviour. Joffe also identified the status quo function of social representations which enable the perpetuation of history through which society continuously make the world seem more familiar and manageable [36]. According to Moscovici, social representations establish an order which enables individuals to familiarise themselves in their material and social world and to master it [37]. Furthermore, social representations enable communication to take place among the members of a community by providing them with a code of social exchange and a code for naming and categorising. The social representations theory has been applied in many different settings including in understanding representations of mental illness, representations of condoms among the youths, and representations of risky sexual behaviour in an era of HIV/AIDS [33, 38, 39]. A social representations approach to the study of VMMC is likely to reveal some of the perceptions and socially constructed meanings of circumcision which may ultimately influence the uptake of circumcision.

## Data and methods

Five focus group discussions with men and women aged between 18 and 49 years were conducted as part of a mixed methods study which collected both qualitative and quantitative data between March and July 2013. Three FGDs were held with men. The first group had 11 men aged between 18 and 40 years while the second group had 10 participants aged between 22 and 48. The third group had 9 participants aged between 19 and 34 years. One FGD was held with 9 female participants aged between 28 and 48 years. The fifth FGD had 10 participants of mixed sex aged between 23 and 34 years. In addition to the five FGDs, five in-depth interviews were also conducted with key informants drawn from the Ministry of Health and Child Care (MoHCC) and other implementing partners. While the entire study collected both qualitative and quantitative data, this paper focuses on the findings obtained from FGDs and in-depth interviews only. The inclusion of men and women aged between 18 and 49 years was premised on the assumption that this group would be more knowledgeable about sex since the majority of them would be sexually active. In addition, the men and women in this broad age group would be influential in the decision to have a son(s) get circumcised for HIV prevention. Recent evidence from the Spear and Shield Project in Zambia has shown that female support is a significant predictor of VMMC uptake among men [40]. The broad age group of 18 to 49 years of both males and females enabled the collection of data that reflects the views of both young and middle-aged participants.

Participants in the FGDs were randomly selected from households that were sampled in high density suburbs of Harare urban except for participants in the mixed sex FGD who were selected using convenience sampling from a local shopping area. Eligible household members who were available during data collection were randomly assigned for either an individual interview (for the quantitative strand) or were asked to take part in a FGD. The FGDs were done with two assistants, one moderating the discussion and the other one recording the discussion on an electronic device. A FGD guide was used to have a well coordinated discussion. The guide solicited respondents’ views on the meaning of circumcision, what people say when they talk about VMMC, people’s perceptions of circumcision and perceived barriers to circumcision uptake. The use of FGDs to collect qualitative data enabled the study to develop an understanding of why men are reluctant to get circumcised and why women are hesitant to encourage men to get circumcised. The FGDs method also allowed participants’ views on certain aspects of VMMC to be questioned by fellow participants [41].

In addition to FGDs, key informant interviews were also conducted with representatives from the Ministry of Health, the Zimbabwe Council of Churches, the Evangelical Fellowship of Zimbabwe, the Zimbabwe National AIDS Council and the Zimbabwe National Family Planning Council. In-depth interviews with key informants were conducted using a guide and were all digitally recorded. The guide for key informants solicited information on how the VMMC upscale programme was started, its implementation challenges, and people’s response to the roll-out of VMMC for HIV prevention. Analysis of qualitative data primarily involved transcribing, coding and categorisation of statements into identified themes. Initially twelve categories or themes were identified during analysis and these were reduced to only five.

The conduct of this study was approved by the Ethics Committee of the College of Humanities of the University of KwaZulu Natal, South Africa. All participants in the study were 18 years and above. In addition, each participant was required to sign a standard Informed Consent Form which was written in English and translated to Shona. In addition to getting ethical clearance, several strategies were adopted to ensure the validity and reliability of the study in line with suggestions by Lincoln and Guba and Shenton [42, 43]. Firstly, the study adopted well tried and tested methodologies of data collection and data analysis. These were further strengthened by using randomly selected participants. Holding several FGDs and in-depth interviews ensured that particular viewpoints were confirmed from different sources. Debriefing sessions between the main researchers and assistants was also critical in ensuring the quality of data.

## Results

### Meanings of circumcision

The promotion of voluntary medical male circumcision (VMMC) has been emphasizing cleanliness as one of the reasons for men to consider getting a circumcision. In a society where the practice of circumcision or discussions surrounding the practice was virtually non-existing prior to the roll-out campaign, the understanding of circumcision in terms of cleanliness is not surprising. For some participants, their definition and understanding of circumcision was synonymous to that of cleanliness. One participant defined circumcision as “getting men cleaned” while another viewed circumcision as the removal of a body part that keeps dirt making it difficult for men to remain clean. These responses can be attributed to the promotion of VMMC for HIV prevention.

*[Circumcision] is getting men cleaned because a circumcised penis gets “fresh air” compared to an uncircumcised one.* (Female FGD participant)

*[Circumcision] is the removal of the foreskin which keeps dirt and without the skin there is no room to keep dirt and it reduces the breeding of bacteria.* (Male FGD participant)

Circumcision was also defined in terms of its protective effect against STIs and HIV. A male participant equated circumcision to wearing gloves when handling fire. It was noted that while you would eventually get burnt while wearing the gloves, the gloves would delay that process. What this means is that circumcision is viewed as affording temporary protection against HIV infection. The general interpretation here is that a circumcised man has the advantage of delayed vulnerability compared to an uncircumcised man. A more radical understanding of VMMC’s protective effect was expressed by other participants who equated circumcision to an *“invisible condom”.*

*[Circumcision] is wearing gloves while handling fire. You will get burnt definitely but that would take time. Sixty percent of the time you will be protected but the other 40 % you will hold direct fire.* (Male FGD participant)

The notion that circumcision is similar to wearing gloves or to having an invisible condom gives the impression that messages of the 60 % protective effect afforded by VMMC toward HIV and other STIs have not been well understood by some. On the other hand, there are indications that some people seem to have a clearer understanding of the protective effect of VMMC. Some participants mentioned that circumcision reduces the risk of getting infected with sexually transmitted infections. VMMC was perceived as *‘second protection”* in the event of a condom breaking. The view of circumcision as second protection supports earlier assertions that circumcision is like a pair of gloves which protects from fire.

*Some are doing it [circumcision] because doctors say it works [for HIV prevention].* (Male FGD participant)

### Circumcision and sexuality

Participants in this study were asked to consider some of the factors which may encourage men to get circumcised. Some male participants, while not doubting the prophylactic nature of VMMC, noted that there are men who would seriously consider getting a circumcision because of the belief that circumcision improves sexual performance. There is a belief among men that a circumcised man is more likely than an uncircumcised one to satisfy a woman. It is not clear where this belief emanates from but it appears to be strong among men. Some participants expressed the view that a circumcised penis is likely to have a stronger erection than an uncircumcised one because the foreskin hinders a full erection. The strong erection was then associated with multiple orgasms for female partners.

*A circumcised penis is more satisfying.* (Female FGD participant)

*It [circumcision] helps the penis to be more erect than when it has the foreskin and leads to more orgasms for women.* (Male FGD participant)

The sex motive was also mentioned by other respondents who noted that the promotion of VMMC has unveiled an opportunity for some ‘adventurous’ men to explore different sexual experiences with a circumcised penis. It was mentioned that some men may just get circumcised out of curiosity to feel the difference between sex with an uncircumcised penis and that with a circumcised one. The sexual adventures do not only end with the curiosity to experience a different sex, but also to be able to lure potential sexual partners. It was highlighted that some men were simply following advice from an earlier advertisement which implored men to get circumcised because ‘beautiful girls love circumcised men’.

*They [some men] are just experimenting on how it [sex] feels after circumcision.... The message on the advert says beautiful girls love men who are circumcised so men are doing it [getting circumcised] to be the ladies’ target.* (Key Informant)

There are evidently divergent views from the participants in this study on why men may consider getting a circumcision. What is clear though is that HIV prevention is only one among a plethora of justifications for getting circumcised. Some of these reasons may actually be at odds with the actual reasons behind the promotion of VMMC. There were participants who felt that this ability of circumcision to give men ‘more power’ in bed was not good because it would lead to promiscuity thus defeating the purpose for which VMMC is being promoted- HIV prevention.

*It’s [circumcision] not right because its giving a person desire and courage to sleep around and cause more spread of HIV.* (Male FGD participant)

*It [circumcision] is said to increase sexual desire so much that a single woman won’t satisfy you so you have to hit here and there.* (Male FGD participant)

Some respondents disagreed with the view that circumcision increases sexual desire. They noted that circumcision in fact causes a loss of sexual desire as illustrated by references to the penis becoming “cold” because its “warming cover” would have been removed. The reference to a ‘cold’ penis implies a loss of sexual desire or ability to achieve a good erection. Fears of losing fertility due to circumcision were also mentioned. There were also fears that getting circumcised might lead to marriage breakdown due to the prolonged healing period before resumption of intercourse. Because of this, some suggested that circumcision be promoted among young children who do not yet have sexual obligations to fulfil.

*A married man cannot get circumcised because he risks losing his wife, she will run away. It is difficult to sleep next to each other then not have sex…circumcise kids below 12 years who haven’t ‘tasted’ sex at all, that way it will work.* (Male FGD participant)

### Condom use after circumcision

The issue of condom use after circumcision is a topical issue in the current discourse on VMMC promotion for HIV prevention. This study asked respondents to give their views or what they hear people say when they talk about condom use after getting a circumcision. Several participants expressed the view that it would be unnecessary to use a condom after getting circumcised. One participant remarked that a circumcised penis would tear a condom therefore there is no need to wear one (Male participant, age range 23–34 years in mixed sex FGD). This notion that a circumcised penis ‘tears’ a condom seem to emanate from a misunderstanding of the keratinisation process that takes place after circumcision. Some participants felt that the hardening of the surface skin of the penis reduced sensitivity during intercourse and thus using a condom would further reduce the pleasure for a man. Another participant expressed similar sentiments by noting that a circumcised penis is exposed so putting a condom will make one feel pain while another male participant likened wearing a condom to being *“over-dressed for the party”,* that is, having a circumcision and a condom all for HIV prevention. Another participant highlighted that messages promoting voluntary medical male circumcision had given an impression that your chances of getting infected with HIV during unprotected sex are only 40 % and this has led to more people opting for unprotected sex. Some respondents even questioned the efficacy of male circumcision given that one has to use condoms even after getting a circumcision.

*The message of reducing chances of not getting the virus has led to more unprotected sex paying less attention to condoms. Why would someone wear gumboots (condoms) when you are already ‘sorted’ (protected)?* (Male FGD participant)

*The advantages [of male circumcision] are not clear and convincing. Why get cut and still need condoms afterwards, what’s the point?* (Male FGD participant)

### Beliefs associated with circumcision

The views expressed by FGD participants portrayed circumcision as an alien culture or part of a foreign religion. Respondents noted that circumcision is not a common practice in Zimbabwe, except among the Vamwenye and the Chewa who are considered immigrants from Malawi. They also pointed out that the practice is done by Moslems and Jewish people as part of their religion. A male participant in the 22–48 years male only FGD remarked that:

*Some people believe that it’s [circumcision] not part of the culture and ancestors don’t like it that is why there are no rains coming down in Zimbabwe.*

In addition to circumcision being viewed as an alien culture, participants in the FGDs also expressed fears that the removed foreskin may be used in satanic rituals. Respondents mentioned that there are entrenched fears of unwillingly and/or unwittingly joining satanic cults by getting circumcised. These fears seem to emanate from endless rumours and stories about Satanism that circulate within the community. Owing to these fears, it was mentioned that people are not sure of what will happen to their foreskins once they are circumcised.

A male participant noted that there are stories that circulate in the community about foreskins that are sold at exorbitant prices and exported to South Africa. He remarked that:

*We hear that the removed foreskins are exported to South Africa where they are used in money rituals or to make skin lotions for women.* (Male FGD participant)

### Circumcision, stigma and disability

In addition to the association of circumcision to ritualistic ceremonies and Satanism, respondents also highlighted that circumcision is associated with illness and disability which may lead to ridicule in a society where the practice is still relatively new. The concomitant stigma in the community was highlighted as deterrent to the eventual uptake of VMMC. A circumcised man in the discussion highlighted that when he got circumcised he was unable to put on a trousers for some days and his friends ridiculed him because they assumed that had an STI.

*When you get circumcised, it will be painful to wear underclothes and sometimes you have to wear a dress like a woman to avoid that pain. Sometimes your friends might laugh at you when they see you walking slowly to avoid hurting the wound and they say you have “sick” (a sexually transmitted infection).* (Male FGD participant)

Another source of stigma that was expressed by a few of the respondents is that in some circles circumcised men are viewed as disabled or as having a disability. This view appears to emanate from a likening of circumcision to a condition in which a person is born with a retracted foreskin (*“nzvonyo”* in some Shona dialects) which was viewed by some as a form of disability.

*Men are shy. Circumcision is still a new practice and also uncommon and might attract ridicule among peers in some circles.* (Female Key Informant)

During one of the FGD with men aged between 19 and 34 years, it was noted that hospitals are not the ideal place to do the circumcisions because of their association with sickness and death. One of the centres offering VMMC services is at Spilhouse which is adjacent to Harare Hospital, one of the largest public referral hospitals in the country. Some participants felt that circumcising men at hospitals gives the impression that you are already ill and therefore need medical attention. It was also highlighted that when you get to that hospital site, you get memories of friends and relatives who walked into the hospital looking healthy but were carried away dead. Respondents further noted that the association between circumcision and illness is further compounded by the requirement to undergo HIV testing before getting circumcised. In a country riddled with high HIV prevalence and AIDS related deaths, it has become routine practice for medical personnel to request patients to undergo HIV testing in order to eliminate HIV as a possible cause of illness.

### Women’s involvement in VMMC

This study sought participants’ views on the role that women can play in the promotion of VMMC for HIV prevention. One view that was presented during the discussions is that women have persuasive power and thus can influence their sons and husbands to get circumcised. In addition to women having the power to persuade men to get circumcised, it was also felt that women should take an active role in promoting VMMC because they are direct beneficiaries of the programme through reduced chances of getting cervical cancer and through improved sexual satisfaction.

Other participants, particularly women, were sceptical about their active involvement in the promotion of circumcision citing fear and communication challenges. Women participants expressed fear that the promotion of VMMC may transmit the wrong signals thus leading some men to become reckless and promiscuous. They also noted that because of the belief that circumcision boosts a man’s sexual appetite, they feared that they would not be able to satisfy their partners thus increasing the likelihood of them seeking sexual gratification elsewhere. A female participant in the female only FGD noted the following:

*Women fear that if their husbands are circumcised they will behave like bulls and the women will not be able to satisfy them sexually, it’s inviting problems for yourself.* (Female FGD participant)

Women also pointed out that they could not encourage their partners to get circumcised on the pretext that circumcision improves sexual performance because they feared that men would accuse them of infidelity. In particular, a man would demand to know how the wife/partner got to know that a circumcised man performs better in bed. A female participant in the mixed gender FGD remarked that:

*Some men will be problematic because they would want to know how their wives got to know that a circumcised penis is better than an uncircumcised one.* (Female FGD participant)

Besides encouraging men to get circumcised, it was suggested that women can also help in the process by going away from home to allow the man to heal without a possibility of engaging in sexual intercourse before the recommended healing time of six weeks. It was mentioned that 6 weeks is a very long time to spend without having sex and thus the temptation is greater when a woman is readily available.

*For men to heal better and faster women should leave the house to avoid sharing the same bed so that men are not [sexually] aroused.* (Male FGD participant)

## Discussion and conclusion

The purpose of this paper was to contribute to the ongoing discussion about the inclusion of male circumcision as an additional HIV prevention strategy through an evaluation of the social representations of VMMC for HIV prevention. The findings of this study have uncovered representations which are critical in understanding some of the factors influencing the uptake of VMMC. The scaling up of voluntary medical male circumcision was started in 2009 but only 170, 000 men had volunteered for circumcision as at September 2013 against an expected target of 1.9 million. This slow rate of uptake cannot be explained by a general lack of knowledge because there is evidence to suggest that men and women in Zimbabwe are knowledgeable about VMMC [12]. However, the levels of knowledge of VMMC need to be continuously monitored through the development of more rigorous items instead of relying on questions that simply ask respondents whether they have ever heard of VMMC or not. A recent study in Malawi showed that while a large proportion of men and women believed that VMMC reduces the chances of getting infected with HIV for men, they also erroneously believed that it provided protection from HIV for women [44].

This study has established that circumcision is viewed as alien to the general cultural practices of the local people. This finding is similar to the views expressed by older men in Turkana County, Kenya who spoke of circumcision as belonging to other cultures [45]. Similar findings were also reported in rural Zimbabwe [46, 47]. Also similar to views expressed in Turkana is the perception that circumcision might invite stigma from peers. Participants in a study by Moyo et al*.* in rural Zimbabwe expressed fear of stigmatisation to the point that they reported fearing using public facilities [47]. The view that circumcision is seen as a form of disability needs further investigation because it is likely to impact negatively on the uptake of male circumcision for HIV prevention. There is need to understand the dynamics surrounding this representation and thus design communications that address these conceptualisations. It has been suggested that messages promoting VMMC should focus on the normality of the circumcised penis [45].

This study has also shown that there is a need to understand how myths and beliefs surrounding circumcision can impact the uptake of voluntary medical male circumcision for HIV prevention. Myths that link circumcision to dark and evil forces such as Satanism and witchcraft may be difficult to dispel without a clear understanding of the source of these beliefs. It is suggested that programmes that promote circumcision should involve traditional/spiritual authorities/healers who can be at the forefront of dissuading such myths and beliefs. It is also suggested that there is need for communications that show transparency in how the entire circumcision experience is handled from the point when a person walks into a circumcision clinic up to the point when the foreskins are disposed.

The association between circumcision and sexual pleasure was highlighted as a major point of discussion. This issue has also been raised in literature but it appears that there is no general agreement between scholars. It has been reported that circumcised men are more likely to cite significantly reduced penile sensitivity, increased ejaculatory problems and greater dissatisfaction with their sex lives than their uncircumcised counterparts [48]. It has also been reported that partners of circumcised men are more likely to report vaginal dryness and difficulty reaching orgasm than women with intact partners [49]. Other studies have concluded that male circumcision is unlikely to adversely affect sexual performance [50]. Given the views expressed by participants in this study, there is need for communications that address this issue. Similarly worrying are the views expressed on condom use after circumcision. However, these views are generally addressed during the initial counselling process.

This study also explored the possible contribution of women in the roll-out of voluntary male circumcision. The findings suggest that the discussion of circumcision as an HIV prevention strategy within intimate relationships maybe fraught with suspicions of infidelity. On one hand, men may be suspicious of a woman’s suggestion that he be circumcised because that may be interpreted as suggesting that man is not good in bed or that the woman has had intercourse with a circumcised man and has thus developed preference for a circumcised penis. On the other hand, men cannot also raise the issue for discussion because the woman would suspect that the man intends to get HIV protection from extra marital unions. Thus, circumcision among men in long-term intimate relationships is likely to suffer the same fate as that of the condom which became a symbol of mistrust and infidelity in marital unions.

In conclusion, the views expressed by participants in this study point to a need to continue designing communications that directly address myths and misconceptions about male circumcision. In particular, there is need to address the misconception that VMMC is for other cultures, that circumcision is a form of disability and also issues surrounding circumcision and sexuality. The above conclusions from this study should be understood in the context of a number of limitations. Firstly, the study findings and conclusions are based on only five FGDs. These are too few to make sweeping generalisations. Furthermore, the study was conducted using a sample of urban participants only. This naturally excluded potentially variable views of people who reside in other contexts.

## References

[1] Auvert B, Taljaard D, Lagarde E, Sobngwi-Tambekou J, Sitta R, Puren A (2005). Randomized, controlled intervention trial of male circumcision for reduction of HIV infection risk: the ANRS 1265 Trial. PLoS Med.

[2] Gray RH, Kigozi G, Serwadda D, Makumbi F, Watya S, Nalugoda F, Kiwanuka N, Moulton LH, Chaudhary MA, Chen MZ, Sewankambo NK, Wambwire-Mangen F, Bacon MC, Williams CFM, Opendi P, Reynolds SJ, Laeyendecker O, Quinn TC, Wawer MJ (2007). Male circumcision for HIV prevention in men in Rakai, Uganda: a randomised trial. Lancet.

[3] Bailey RC, Moses S, Parker CB, Agot K, Maclean I, Krieger JN, Williams CFM, Campbell RT, Ndinya-Achola A (2007). Male circumcision for HIV prevention in young men in Kisumu, Kenya: a randomised controlled trial. The Lancet.

[4] Price LB, Liu CM, Johnson KE, Aziz M, Lau MK, Bowers J, Ravel J, Keim PS, Serwadda D, Wawer MJ, Gray RH (2010). The effects of circumcision on the penis microbiome. PLoS One.

[5] Muula AS, Prozesky HW, Mataya RH, Ikechebelu JI (2007). Prevalence of complications of male circumcision in Anglophone Africa: a systematic review. BMC Urol.

[6] Okeke LI, Asinobi AA, Ikuerowo OS (2006). Epidemiology of complications of male circumcision in Ibadan, Nigeria. BMC Urol.

[7] Mattson C, Campbell RT, Bailey RC, Ago K, Ndinya-Achola JO, Moses S (2008). Risk Compensation Is Not Associated with Male Circumcision in Kisumu Kenya: A Multi-Faceted Assessment of Men Enrolled in a Randomized Controlled Trial. PLoS One.

[8] McAllister RG, Travis JW, Bollinger D, Rutiser C, Sundar V (2008). The cost to circumcise Africa. Int J Men’s Health.

[9] Herman-Roloff A, Otieno N, Ndinga-Achola J, Bailey RC (2011). Acceptability of medical male circumcision among uncircumcised men in Kenya one year after the launch of the National Male Circumcision program. PLoS One.

[10] Godlonton S, Muthuli A, Thorton R (2011). Behavioral response to information? Circumcision, information and HIV prevention. BREAD working paper No 313.

[11] Tobian AA, Gray RH, Quinn TC (2010). Male circumcision for the prevention of acquisition and transmission of sexually transmitted infections: the case for neonatal circumcision. Arch Pediatr Adolesc Med.

[12] Hatzold K, Mavhu W, Jasi P, Chatora K, Cowan FM, Taruberekera N, Mugurungi O, Ahanda K, Njeuhmeli E (2014). Barriers and Motivators to Voluntary Medical Male Circumcision Uptake among Different Age Groups of Men in Zimbabwe: Results from a Mixed Methods Study. PLoS One.

[13] Zimbabwe National Statistics Agency (ZIMSTAT) and ICF International (2012). Zimbabwe Demographic and Health Survey 2010–11.

[14] Mavhu W, Buzduga R, Langhaug LF, Hatzold K, Benedikt C, Sherman J, Laver SM, Mundida O, Woelk G, Cowan FM (2011). Prevalence and factors associated with knowledge of and willingness for male circumcision in rural Zimbabwe. Trop Med Int Health.

[15] Halperin DT, Fritz K, Mcfarland W, Woelk G (2005). Acceptability of adult male circumcision for sexually transmitted disease and HIV prevention in Zimbabwe. Sex Transm Dis.

[16] Bailey R, Muga R, Poulussen R, Abicht H (2002). The acceptability of male circumcision to reduce HIV infections in Nyanza Province, Kenya. AIDS Care.

[17] Scott BE, Weiss HA, Viljoen JI (2005). The acceptability of male circumcision as an HIV intervention among a rural Zulu population, KwaZulu-Natal. South Africa AIDS Care.

[18] Lissouba P, Taljaard D, Rech D, Dermaux-Msimang V, Legeai C, Lewis D, Singh B, Puren A, Auvert B (2011). Adult male circumcision as an intervention against HIV: An operational study of uptake in a South African community (ANRS 12126). BMC Infect Dis.

[19] Lukobo MD, Bailey RC (2007). Acceptability of male circumcision for prevention of HIV infection in Zambia. AIDS Care.

[20] Perera CL, Bridgewater FH, Thavaneswaran P, Maddern GJ (2010). Safety and efficacy of nontherapeutic male circumcision: a systematic review.*The*. Ann Fam Med.

[21] Weiss HA, Larke N, Halperin D, Schenker I (2010). Complications of circumcision in male neonates, infants and children: a systematic review. BMC Urol.

[22] Longmore MA (1998). Symbolic interactionism and the study of sexuality. J Sex Res.

[23] Karlyn AS (2005). Sexual identity, risk perceptions and AIDS prevention scripts among young people in Mozambique.

[24] Farrar, L. ‘Why Men Don’t Use Condoms in a HIV Epidemic: Understanding Condom Neglect through Condom Symbology’, Reinvention: an International Journal of Undergraduate Research, BCUR/ICUR 2013 Special Issue, 2013. http://www2.warwick.ac.uk/fac/cross_fac/iatl/reinvention/issues/bcur2013specialissue/farrar/. Accessed 01 September 2014.

[25] Mbiti J (1997). Introduction to African Religion.

[26] Mshana G, Wambura M, Mwanga J, Mosha J, Mosha F, Changalucha J (2011). Traditional male circumcision practices among the Kurya of North-eastern Tanzania and implications for national programmes. AIDS Care: Psychological and Socio-medical Aspects of AIDS/HIV.

[27] Meissner O, Buso DL (2007). Traditional male circumcision in the Eastern Cape--scourge or blessing?. S Afr Med J.

[28] Vincent L (2008). ‘Boys will be boys’: Traditional Xhosa male circumcision, HIV and sexual socialisation in contemporary South Africa. Culture, Health & Sexuality.

[29] Lawlor R (1991). Voices of the First Day: Awakening in the Aboriginal Dreamtime.

[30] Hosken F (1994). The Hosken report: Genital and sexual mutilation of females.

[31] Hellsten SK (2004). Rationalising circumcision: From tradition to fashion, from public health to individual freedom: Critical notes on cultural persistence of the practice of genital mutilations. J Med Ethics.

[32] Moscovici S, Forgas JP (1981). On social representations. Social cognition: Perspectives in everyday understanding.

[33] Winskell K, Obyerodhyambo O, Stephenson R (2011). Making sense of condoms: Social representations in young people’s HIV-related narratives from six African countries. Soc Sci Med.

[34] Maurya AS (2009). Integrating Social Representations Theory and Bio-psychosocial Model for intervention in Mental Health. J Indian Acad Appl Psychol.

[35] Walmsley CJ. Social representation and the study of professional practice. Int J Qual Methods. 2004. 3(4), Article 4. Retrieved 29/11/11 from https://www.ualberta.ca/~iiqm/backissues/3_4/pdf/walmsley.pdf

[36] Joffe H (1996). AIDS research and prevention: A social representational approach. Br J Med Psychol.

[37] Moscovici S (1984). The myth of the lonely paradigm: A rejoinder. Soc Res.

[38] Dixit S (2005). Meaning and explanations of mental illness: A Social Representations Approach. Psychol Dev Soc.

[39] Camargo BV, Bousfield ABS (2009). Social Representations, Risk Behaviours and AIDS. Span J Psychol.

[40] Cook R, Jones D, Redding CA, Zulu R, Chitalu N, Weiss SM. Female Partner Acceptance as a Predictor of Men’s Readiness to Undergo Voluntary Medical Male Circumcision in Zambia: The Spear and Shield Project. AIDS Behav. 2015;1–11. Epub ahead of print 10.1007/s10461-015-1079-x PMC4696907 25931242

[41] Bryman A (2008). Social Research Methods.

[42] Lincoln YS, Guba EG (1985). Naturalistic inquiry.

[43] Shenton AK (2004). Strategies for ensuring trustworthiness in qualitative research projects. Educ Inf.

[44] Maughan-Brown B, Godlonton S, Thornton R, Venkataramani AS. What Do People Actually Learn from Public Health Campaigns? Incorrect Inferences About Male Circumcision and Female HIV Infection Risk Among Men and Women in Malawi. AIDS Behav. 2014;1–8. Epub ahead of print 10.1007/s10461-014-0882-0 25155700

[45] Macintyre K, Andrinopoulos K, Moses N, Bornstein M, Ochieng A, Peacock E, Bertrand J (2014). Attitudes, Perceptions and Potential Uptake of Male Circumcision among Older Men in Turkana County, Kenya Using Qualitative Methods. PLoS One.

[46] Khumalo-Sakutukwa G, Lane T, Van-Rooyen H, Chingono A, Humphries H, Timbe A, Fritz K, Chirowodza A, Morin SF (2013). Understanding and addressing socio-cultural barriers to medical male circumcision in traditionally non-circumcising rural communities in sub-Saharan Africa. Culture Health Sex.

[47] Moyo S, Mhloyi M, Chevo T, and Rusinga O. Men’s attitudes: A hindrance to the demand for voluntary medical male circumcision–A qualitative study in rural Mhondoro-Ngezi, Zimbabwe. Glob Public Health. 2015;1–13. (ahead-of-print) 10.1080/17441692.2015.1006241 25648951

[48] Bensley AG, Boyle GJ, Denniston GC, Hodges FM, Milos MF (2000). Physical, sexual and psychological effects of male infant circumcision: an exploratory survey. Understanding circumcision: a multidisciplinary approach to a multidimensional problem.

[49] O’Hara K, O’Hara J (1999). The effect of male circumcision on the sexual enjoyment of the female partner. BJU Int.

[50] Krieger JN, Mehta SD, Bailey RC, Agot K, Ndinya-Achola JO, Parker C, Moses S (2008). Adult male circumcision: effects on sexual function and sexual satisfaction in Kisumu, Kenya. J Sex Med.

